# First-In-Human Phase I Study of Tinengotinib (TT-00420), a Multiple Kinase Inhibitor, as a Single Agent in Patients With Advanced Solid Tumors

**DOI:** 10.1093/oncolo/oyad338

**Published:** 2024-01-31

**Authors:** Sarina A Piha-Paul, Binghe Xu, Ecaterina E Dumbrava, Siqing Fu, Daniel D Karp, Funda Meric-Bernstam, David S Hong, Jordi A Rodon, Apostolia M Tsimberidou, Kanwal Raghav, Jaffer A Ajani, Anthony P Conley, Frank Mott, Ying Fan, Jean Fan, Peng Peng, Hui Wang, Shumao Ni, Caixia Sun, Xiaoyan Qiang, Wendy J Levin, Brenda Ngo, Qinhua Cindy Ru, Frank Wu, Milind M Javle

**Affiliations:** Department of Investigational Cancer Therapeutics, The University of Texas, MD Anderson Cancer Center, Houston, TX, USA; Department of Medical Oncology, National Cancer Center/Cancer Hospital, Chinese Academy of Medical Sciences and Peking Union Medical College, Beijing, People’s Republic of China; Department of Investigational Cancer Therapeutics, The University of Texas, MD Anderson Cancer Center, Houston, TX, USA; Department of Investigational Cancer Therapeutics, The University of Texas, MD Anderson Cancer Center, Houston, TX, USA; Department of Investigational Cancer Therapeutics, The University of Texas, MD Anderson Cancer Center, Houston, TX, USA; Department of Investigational Cancer Therapeutics, The University of Texas, MD Anderson Cancer Center, Houston, TX, USA; Department of Investigational Cancer Therapeutics, The University of Texas, MD Anderson Cancer Center, Houston, TX, USA; Department of Investigational Cancer Therapeutics, The University of Texas, MD Anderson Cancer Center, Houston, TX, USA; Department of Investigational Cancer Therapeutics, The University of Texas, MD Anderson Cancer Center, Houston, TX, USA; Department of Gastrointestinal Medical Oncology, The University of Texas, MD Anderson Cancer Center, Houston, TX, USA; Department of Gastrointestinal Medical Oncology, The University of Texas, MD Anderson Cancer Center, Houston, TX, USA; Department of Sarcoma Medical Oncology, The University of Texas, MD Anderson Cancer Center, Houston, TX, USA; Department of Thoracic and Head and Neck Medical Oncology, The University of Texas, MD Anderson Cancer Center, Houston, TX, USA; Department of Medical Oncology, National Cancer Center/Cancer Hospital, Chinese Academy of Medical Sciences and Peking Union Medical College, Beijing, People’s Republic of China; Clinical Department, TransThera Sciences (US), Inc., Gaithersburg, MA, USA; Project Management Department, TransThera Sciences (Nanjing), Inc., Nanjing, People’s Republic of China; Clinical Department, TransThera Sciences (Nanjing), Inc., Nanjing, People’s Republic of China; Drug Metabolism and Pharmacokinetics Department, TransThera Sciences (Nanjing), Inc., Nanjing, People’s Republic of China; Clinical Department, TransThera Sciences (Nanjing), Inc., Nanjing, People’s Republic of China; Biology Department, TransThera Sciences (Nanjing), Inc., Nanjing, People’s Republic of China; Clinical Department, CRC Oncology, San Diego, CA, USA; Clinical Department, CRC Oncology, San Diego, CA, USA; Clinical Department, CRC Oncology, San Diego, CA, USA; Project Management Department, TransThera Sciences (Nanjing), Inc., Nanjing, People’s Republic of China; Drug Metabolism and Pharmacokinetics Department, TransThera Sciences (Nanjing), Inc., Nanjing, People’s Republic of China; Department of Gastrointestinal Medical Oncology, The University of Texas, MD Anderson Cancer Center, Houston, TX, USA

**Keywords:** solid tumors, kinase inhibitor, phase I, tinengotinib, clinical study

## Abstract

**Purpose:**

This first-in-human phase I dose-escalation study evaluated the safety, pharmacokinetics, and efficacy of tinengotinib (TT-00420), a multi-kinase inhibitor targeting fibroblast growth factor receptors 1-3 (FGFRs 1-3), Janus kinase 1/2, vascular endothelial growth factor receptors, and Aurora A/B, in patients with advanced solid tumors.

**Patients and Methods:**

Patients received tinengotinib orally daily in 28-day cycles. Dose escalation was guided by Bayesian modeling using escalation with overdose control. The primary objective was to assess dose-limiting toxicities (DLTs), maximum tolerated dose (MTD), and dose recommended for dose expansion (DRDE). Secondary objectives included pharmacokinetics and efficacy.

**Results:**

Forty-eight patients were enrolled (dose escalation, *n* = 40; dose expansion, *n* = 8). MTD was not reached; DRDE was 12 mg daily. DLTs were palmar-plantar erythrodysesthesia syndrome (8 mg, *n* = 1) and hypertension (15 mg, *n* = 2). The most common treatment-related adverse event was hypertension (50.0%). In 43 response-evaluable patients, 13 (30.2%) achieved partial response (PR; *n* = 7) or stable disease (SD) ≥ 24 weeks (*n* = 6), including 4/11 (36.4%) with *FGFR2* mutations/fusions and cholangiocarcinoma (PR *n* = 3; SD ≥ 24 weeks *n* = 1), 3/3 (100.0%) with hormone receptor (HR)-positive/HER2-negative breast cancer (PR *n* = 2; SD ≥ 24 weeks *n* = 1), 2/5 (40.0%) with triple-negative breast cancer (TNBC; PR *n* = 1; SD ≥ 24 weeks *n* = 1), and 1/1 (100.0%) with castrate-resistant prostate cancer (CRPC; PR). Four of 12 patients (33.3%; HR-positive/HER2-negative breast cancer, TNBC, prostate cancer, and cholangiocarcinoma) treated at DRDE had PRs. Tinengotinib’s half-life was 28-34 hours.

**Conclusions:**

Tinengotinib was well tolerated with favorable pharmacokinetic characteristics. Preliminary findings indicated potential clinical benefit in FGFR inhibitor-refractory cholangiocarcinoma, HER2-negative breast cancer (including TNBC), and CRPC. Continued evaluation of tinengotinib is warranted in phase II trials.

## Teaser

This first-in-human study was conducted to characterize the safety, tolerability, pharmacokinetics, and preliminary efficacy of single-agent tinengotinib in patients with advanced solid tumors.

**Implications for Practice**

This was the first clinical study of tinengotinib (TT-00420), a multi-kinase inhibitor targeting fibroblast growth factor receptors 1-3 (FGFRs 1-3), Janus kinase [JAK] 1/2, vascular endothelial growth factor receptors, and Aurora A/B. Tinengotinib was given to 48 patients with advanced solid tumors and was well tolerated, with favorable pharmacokinetics. Forty-one patients (85.4%) had drug-related side effects, the most common being hypertension (50.0%), diarrhea (33.3%), palmar-plantar erythrodysesthesia syndrome (29.2%), stomatitis (29.2%), nausea (22.9%), and vomiting (20.8%). Preliminary efficacy was reported in patients with FGFR inhibitor-refractory cholangiocarcinoma, hormone-receptor-positive/HER2-negative breast cancer, triple-negative breast cancer, and castrate-resistant prostate cancer. These findings suggest that tinengotinib may be effective for patients with different solid tumors.

## Introduction

Receptor tyrosine kinase signaling is frequently dysregulated in human cancers and contributes to many of the hallmark features of tumor biology, including proliferation, angiogenesis, and immune response evasion.^1^ Selective targeting of these molecular drivers with small-molecule tyrosine kinase inhibitors can produce meaningful clinical benefit, although the effects of many are limited by acquired drug resistance.^2^ Strategies to overcome resistance include combination therapy with 2 agents directed against the original target kinase and the predominant cause of resistance,^1,2^ or single-agent therapy with a kinase inhibitor that simultaneously targets multiple tyrosine kinases.

Tinengotinib (TT-00420) is a small-molecule, spectrum-selective, multiple kinase inhibitor that inhibits kinases involved with mitosis (Aurora A/B), angiogenesis (vascular endothelial growth factor receptors [VEGFRs], fibroblast growth factor receptors [FGFRs] 1, 2, and 3), and tumor cell proliferation and immune activity (Janus kinase [JAK] 1/2; colony-stimulating factor 1 receptor) at low nanomolar concentrations.^3^ In preclinical models, tinengotinib inhibited tumor growth in cell-line-derived and patient-derived xenograft (PDX) models of triple-negative breast cancer (TNBC),^3^ cholangiocarcinoma, bladder cancer, hepatocellular carcinoma, and thyroid cancer.^4^ Tinengotinib also demonstrated good oral bioavailability and dose-dependent exposure following oral administration in rats and dogs, with mechanism-related but manageable toxicities.^5^

This first-in-human study (ClinicalTrials.gov NCT03654547) was conducted to characterize the safety, tolerability, pharmacokinetics, and preliminary efficacy of single-agent tinengotinib in patients with advanced solid tumors.

## Patients and Methods

### Study Design

This 2-part, open-label, phase I study was conducted at the MD Anderson Cancer Center in Texas, USA and the Cancer Hospital Chinese Academy of Medical Sciences in Beijing, China. The initial dose-escalation phase was designed to determine the maximum tolerated dose (MTD), dose-limiting toxicities (DLTs), and dose recommended for dose expansion (DRDE) for tinengotinib. This was followed by a dose-expansion phase, the primary objective of which was to confirm the safety and tolerability of tinengotinib at the DRDE. Secondary objectives were to characterize the pharmacokinetics and preliminary efficacy of tinengotinib.

Dose escalation was guided by an adaptive Bayesian logistic regression model using the Escalation with Overdose Control principle.^6,7^ Study data (including DLTs, grade 2 adverse events during cycle 1, and pharmacokinetic data) were reviewed by the sponsor and trial investigators at each dose level and used to guide dose-escalation decisions. MTD was based on the Bayesian model, but with consideration given to available safety and tolerability data. DRDE was based on an evaluation of all available data (safety, clinical activity, and pharmacokinetics).

The study was conducted in accordance with the International Conference on Harmonisation Harmonised Tripartite Guidelines for Good Clinical Practice, with applicable local regulations, and the Declaration of Helsinki. The protocol was reviewed and approved by independent ethics committees at each study site. All patients were required to provide written informed consent prior to enrollment.

### Patient Selection

Eligible patients had histologically or cytologically confirmed locally advanced or metastatic solid tumors. For the dose-expansion phase, preferred indications were cholangiocarcinoma harboring *FGFR2* alterations (all patients with cholangiocarcinoma were considered eligible, regardless of *FGFR2* status), advanced HER2-negative breast cancer (ie, hormone receptor [HR]-positive/HER2-negative or TNBC defined according to American Society of Clinical Oncology/College of American Pathologists guidelines^8^), and selected advanced solid tumors including urothelial carcinoma, gallbladder cancer, gastric cancer, and small cell lung cancer. Patients were 18-75 years of age with adequate organ function, an Eastern Cooperative Oncology Group performance status ≤1, and at least one measurable lesion defined by Response Evaluation Criteria in Solid Tumors (RECIST), version 1.1.^9^ Detailed eligibility criteria are outlined in Supplementary Methods.

### Drug Treatment

Tinengotinib was administered orally (capsule formulation) once daily on a 28-day cycle until documented progression, unacceptable toxicity, or withdrawal of consent. Provisional tinengotinib dose levels during the dose-escalation phase were 1, 3, 5, 8, 10, 12, 15, 18, and 20 mg. After determination of the DRDE, the dose-expansion phase was opened to further characterize safety and pharmacokinetics, and to assess preliminary efficacy.

### Assessments

Molecular screening of tumor samples prior to study entry was not performed and there was no confirmatory testing after study entry. With the exception of an exploratory biomarker analysis in the dose-expansion phase (described later), biomarker data were collected retrospectively from medical records when available.

Routine safety assessments of adverse events, laboratory tests (hematology, coagulation, biochemistry, and urinalysis), vital signs, and 12-lead electrocardiograms were performed. Adverse events were graded according to the National Cancer Institute Common Terminology Criteria for Adverse Events (CTCAE), version 5.0. A DLT was defined as an adverse event or abnormal laboratory value assessed as unrelated to disease, disease progression, intercurrent illness, or concomitant medications, that occurred ≤28 days following the first dose of tinengotinib, and that met any of the protocol-defined criteria for DLT (Supplementary Table S1).

Tumor measurements were obtained at baseline and every 2 cycles (8 weeks) using computerized tomography or magnetic resonance imaging until disease progression or study withdrawal. Tumor responses were determined by local investigator assessment per RECIST, version 1.1.^9^ Confirmed responses were those that were confirmed within 4-8 weeks of the initial response.

### Pharmacokinetic Assessments

Blood samples for the pharmacokinetic evaluation of tinengotinib were collected on pharmacokinetic lead-in day 1 (single dose) and day 28 of cycle 1 (steady state). Single-dose and steady-state pharmacokinetic parameters (area under the concentration-time curve from 0 to 24 hours [AUC_0-24_], maximum concentration [C_max_], time to C_max_ [T_max_], and elimination half-life [t_1/2_]) were estimated when feasible. Plasma concentrations of tinengotinib were measured by validated liquid chromatography-tandem mass spectrometry assay (limit of quantitation, 0.05 ng/mL).

### Statistical Considerations

Dose-escalation sample size was determined by dose levels and emerging toxicities. Per protocol, a minimum of 21 patients was required for enrollment during dose escalation and 6 evaluable patients had to be treated at the dose level declared as the DRDE. Descriptive statistics were used for safety, pharmacokinetic, and efficacy data as outlined in the protocol and as specified in the statistical analysis plan. Pharmacokinetic parameters were estimated using Phoenix WinNonlin version 7.0 or higher (Certara USA, Inc., Princeton, NJ). All other statistical analyses were performed with SAS software version 9.4 (SAS Institute Inc., Cary, NC). The cutoff date for all analyses was September 9, 2022.

### Exploratory Biomarker and Pharmacodynamic Assessments

#### Biomarker Analysis

An exploratory biomarker analysis was performed in patients enrolled in the dose-expansion cohort only. When feasible, archival tumor tissue (baseline), fresh tumor biopsy, and/or blood samples were collected at baseline, day 1/cycle 3, and at disease progression. For patients with cholangiocarcinoma, assays included next-generation sequencing for *FGFR2* alterations in tumor or plasma, and measurement of plasma FGF-23 levels, increases in which represent FGFR inhibition.

#### Subcutaneous Xenograft Models

To validate its effectiveness in *FGFR2* fusion-driven cholangiocarcinoma in vivo, tinengotinib was administered orally at 15 mg/kg in the CC6204 *FGFR2-BICC1* gene fusion PDX model. Tumor fragments of a PDX model of intrahepatic cholangiocarcinoma with the *FGFR2-BICC1* fusion were implanted subcutaneously in female BALB/c nude mice. Test animals were dosed as indicated and relative tumor volume and tumor growth inhibition (TGI) were calculated. Studies were conducted at Crown Bioscience International (San Diego, CA) according to a protocol approved by the Institutional Animal Care and Use Committee as previously described.^3^

#### ELISA Assay for Cellular phosphorylated FGFR2

Ex vivo pharmacodynamic analysis of phosphorylated FGFR2α inhibition was previously established as a reliable biomarker for predicting clinical efficacy in the FIGHT-101 trial.^10^ We therefore assessed FGFR2 phosphorylation in the KATO III cell line following treatment with tinengotinib. Cells were cultured in healthy donor plasma spiked with serial dilutions of tinengotinib. Cellular phosphorylated FGFR2 was determined using the commercial enzyme-linked immunosorbent assay (ELISA) kit (R&D Systems) according to the manufacturer’s instructions. Pemigatinib was included as a positive control^10^ to indicate if the tinengotinib analysis was reliable.

## Results

### Patient Characteristics

Between January 8, 2019 and May 13, 2021, 48 patients were enrolled and received tinengotinib (dose escalation, *n* = 40; dose expansion, *n* = 8; DRDE, *n* = 20), of whom 43 (DRDE, *n* = 18) were evaluable for tumor response (Supplementary Fig. S1). Patient characteristics at baseline are presented in Table 1, and by dose level in Supplementary Table S2. Patients were predominantly White (68.8%) with a median age of 56.8 years (range, 25-79). The most common primary tumor types were cholangiocarcinoma (*n* = 14; 29.2%) and HER2-negative breast cancer (*n* = 9; 18.8%), including 6 patients with TNBC and 3 patients with HR-positive/HER2-negative breast cancer. Overall, 70.8% of patients had received 3 or more prior systemic anticancer therapies.

**Table 1.** Baseline patient demographics and characteristics (full analysis set).

	Dose escalation	Dose expansion (12 mg)	Total
	(*n* = 40)	(*n* = 8)	(*n* = 48)
Median (range) age, years	57.4 (28‒79)	44.6 (25‒73)	56.8 (25‒79)
<60, n (%)	23 (57.5)	6 (75.0)	29 (60.4)
≥60, n (%)	17 (42.5)	2 (25.0)	19 (39.6)
Sex, n (%)			
Male	16 (40.0)	6 (75.0)	22 (45.8)
Female	24 (60.0)	2 (25.0)	26 (54.2)
Race, n (%)			
White	26 (65.0)	7 (87.5)	33 (68.8)
Black or African American	6 (15.0)	0	6 (12.5)
Asian	6 (15.0)	0	6 (12.5)
Other	2 (5.0)	1 (12.5)	3 (6.3)
ECOG performance status, n (%)			
0	8 (20.0)	1 (12.5)	9 (18.8)
1	32 (80.0)	7 (87.5)	39 (81.3)
Primary tumor site, n (%)			
Liver	7 (17.5)	3 (37.5)	10 (20.8)
Breast	8 (20.0)	1 (12.5)	9 (18.8)
Colon	1 (2.5)	1 (12.5)	2 (4.2)
Lung	2 (5.0)	0	2 (4.2)
Esophagus	2 (5.0)	0	2 (4.2)
Ovary	2 (5.0)	0	2 (4.2)
Salivary gland	2 (5.0)	0	2 (4.2)
Soft-tissue sarcoma	2 (5.0)	0	2 (4.2)
Other	14 (35.0)	3 (37.5)	17 (35.4)
Prior therapies, n (%)	40 (100)	8 (100)	48 (100)
Radiation	22 (55.0)	2 (25.0)	24 (50.0)
Surgery	40 (100)	8 (100)	48 (100)
Anticancer medication	39 (97.5)	8 (100)	47 (97.9)
Chemotherapy	37 (92.5)	8 (100)	45 (93.8)
Hormonal therapy	5 (12.5)	1 (12.5)	6 (12.5)
Immunotherapy	17 (42.5)	1 (12.5)	18 (37.5)
Targeted therapy	13 (32.5)	2 (25.0)	15 (31.3)
Other	2 (5.0)	0	2 (4.2)
Lines of therapy, n (%)			
0	1 (2.5)	0	1 (2.1)
1	6 (15.0)	4 (50.0)	10 (20.8)
2	2 (5.0)	1 (12.5)	3 (6.3)
3	13 (32.5)	0	13 (27.1)
≥4	18 (45.0)	3 (37.5)	21 (43.8)

Abbreviation: ECOG, Eastern Cooperative Oncology Group.

Although biomarker data collection was not mandatory and *FGFR* mutation status did not need to be determined before inclusion in the study, 10 of the 14 patients with cholangiocarcinoma had historical next-generation sequencing data. These were *FGFR2*-*KCNH7* fusion, *FGFR2* N549K mutation; *FGFR2*-*AHCYL*1.F17A2 fusion; *FGFR2* C382R mutation; *FGFR2* F17S6, N549H, and N549K mutations; *FGFR2* fusion; *FGFR2* rearrangement, and *TP53* and *BAP1* mutations; FGFR2-*DNAJC12* fusion; *FGFR2* D101V fusion; *FGFR2*-*TACC2*.F17T11 fusion; and *FGFR2*-*CCDC6*.F17C2 fusion.

### Dose Escalation

During dose escalation, sequential patient cohorts received tinengotinib 1 mg (*n* = 1), 3 mg (*n* = 1), 5 mg (*n* = 4), 8 mg (*n* = 10), 10 mg (*n* = 6), 12 mg (*n* = 12), and 15 mg (*n* = 6). Three DLTs were observed at the 8 mg dose (grade 3 palmar-plantar erythrodysesthesia syndrome, *n* = 1) and at the 15 mg dose (grade 3 hypertension, *n* = 2; Supplementary Table S3). There were no other DLTs at the 12 mg dose during the dose-expansion phase. The MTD for tinengotinib was not reached based on the Bayesian model; however, based on the evaluation of safety, pharmacokinetic, and efficacy data, a DRDE of 12 mg once daily was selected for patients with advanced solid tumors.

### Safety and Tolerability

The median duration of treatment with tinengotinib across all dose levels was 2.9 months (range, 0.5‒14.8). Among the 48 patients who received tinengotinib and were evaluable for safety, 41 patients (85.4%) had study drug-related treatment-emergent adverse events (TEAEs; Table 2; Supplementary Table S4). The most common drug-related TEAEs were hypertension (50.0%), diarrhea (33.3%), palmar-plantar erythrodysesthesia syndrome (29.2%), stomatitis (29.2%), nausea (22.9%), and vomiting (20.8%). Twenty-one patients (43.8%) had drug-related grade 3 TEAEs, including hypertension (27.1%), stomatitis (4.2%), diarrhea (2.1%), palmar-plantar erythrodysesthesia syndrome (2.1%), and nausea (2.1%). No drug-related grade 4 or 5 TEAEs were reported. Hypertension was manageable by dose modifications and/or concomitant antihypertensive medication; no patients permanently discontinued study treatment because of drug-related hypertension. An exposure-response model predicted that the risk of grade ≥3 hypertension was uniform and consistent (<25%) for all doses (Supplementary Fig. S2). Seven patients (14.6%) had drug-related serious adverse events (hypertension, *n* = 1; nausea and hypertension, *n* = 1; anemia and gastrointestinal hemorrhage, *n* = 1; mucosal inflammation, *n* = 1; gastrointestinal infection, *n* = 1; cerebrovascular accident, *n* = 1; palmar-plantar erythrodysesthesia syndrome, *n* = 1). Dose modifications (dose adjustment or temporary interruption) were required because of drug-related TEAEs in 25 patients (52.1%), most commonly hypertension (22.9%). Drug-related TEAEs leading to permanent discontinuation of tinengotinib were documented in 6 patients (6.3%): 1 case of grade 3 palmar-plantar erythrodysesthesia syndrome (8 mg); 2 cases of grade 3 hypertension, and 1 case each of grade 2 nausea, grade 2 vomiting, grade 3 cerebrovascular accident, and grade 2 palmar-plantar erythrodysesthesia syndrome (10 mg); and grade 1 retinal vein occlusion (12 mg).

**Table 2.** Summary of study drug-related TEAEs (safety set).

	Dose escalation (*n* = 40)		Dose expansion (12 mg, *n* = 8)		Total (*n* = 48)	
	Any grade	Grade ≥3	Any grade	Grade ≥3	Any grade	Grade ≥3
Study drug-related TEAE	33 (82.5)	16 (40.0)	8 (100)	5 (62.5)	41 (85.4)	21 (43.8)
Study drug-related serious TEAE	5 (12.5)	0	2 (25.0)	0	7 (14.6)	0
Common study drug-related TEAE^a^						
Hypertension	19 (47.5)	9 (22.5)	5 (62.5)	4 (50.0)	24 (50.0)	13 (27.1)
Diarrhea	15 (37.5)	1 (2.5)	1 (12.5)	0	16 (33.3)	1 (2.1)
Palmar-plantar erythrodysesthesia syndrome	10 (25.0)	1 (2.5)	4 (50.0)	0	14 (29.2)	1 (2.1)
Stomatitis	9 (22.5)	2 (5.0)	5 (62.5)	0	14 (29.2)	2 (4.2)
Nausea	11 (27.5)	1 (2.5)	0	0	11 (22.9)	1 (2.1)
Vomiting	10 (25.0)	0	0	0	10 (20.8)	0
Decreased appetite	5 (12.5)	0	2 (25.0)	0	7 (14.6)	0
Blood thyroid-stimulating hormone increased	4 (10.0)	0	3 (37.5)	0	7 (14.6)	0
Proteinuria	6 (15.0)	0	0	0	6 (12.5)	0
Alanine aminotransferase increased	4 (10.0)	0	1 (12.5)	0	5 (10.4)	0
Aspartate aminotransferase increased	4 (10.0)	0	1 (12.5)	0	5 (10.4)	0
Dry mouth	5 (12.5)	0	0	0	5 (10.4)	0
Myalgia	5 (12.5)	0	0	0	5 (10.4)	0

Data are presented as No. (%).

Abbreviation: TEAE, treatment-emergent adverse event.

^a^Defined as any grade study drug-related TEAE occurring in ≥10% of the total study population. No grade 4 or 5 study drug-related TEAEs were reported.

### Antitumor Activity

**Figure 1.**
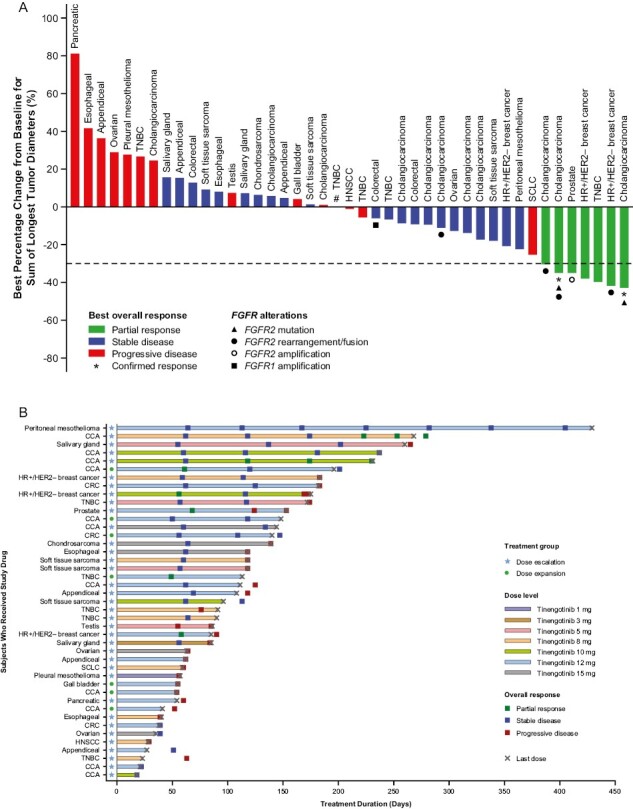
(**A**) Waterfall plot depicting best overall RECIST response^a^ (efficacy analysis set); (**B**) Swimmer plot showing duration of therapy until last dose or data-cut of September 9, 2022 (efficacy analysis set). ^a^Forty-three of 48 patients had at least one post-baseline scan and were evaluable for response. Dotted line shows 30% decrease in tumor size by RECIST version 1.1. ^#^Patient had no target lesions at baseline and their best overall response was SD. Abbreviations: CCA, cholangiocarcinoma; CRC, colorectal; HER2−, human epidermal growth factor 2-negative; HNSCC, head and neck squamous cell carcinoma; HR+, hormone receptor-positive; RECIST, Response Evaluation Criteria in Solid Tumors; SCLC, small cell lung cancer; TNBC, triple-negative breast cancer.

Forty-three patients received at least one dose of study drug and had at least one post-baseline scan (dose escalation, *n* = 36; dose expansion, *n* = 7). As summarized in Fig. 1, partial responses (PRs) were observed in 7 patients (16.3%), including 3 patients with cholangiocarcinoma (8, 10, and 12 mg doses), 2 patients with HR-positive/HER2-negative breast cancer (10 and 12 mg doses), 1 patient with TNBC (12 mg dose), and 1 patient with castration-resistant prostate cancer (CRPC; 12 mg dose). Stable disease (SD) was noted in 23 patients (53.3%), including 6 patients (14.0%) with SD lasting ≥24 weeks (Table 3). At the DRDE of 12 mg once daily, a PR was observed in 4 of 18 (22%) evaluable patients (Table 3).

**Table 3.** Patients with SD for ≥ 24 weeks or PR by RECIST v1.1.

	Patient characteristics				Tinengotinib		Best response by RECIST version 1.1
	Cancer type	*FGFR* alterations^a^	Prior systemic therapies, *n*	Previous targeted therapies	Dose, mg/day	Duration of treatment, weeks	
1001-25	Cholangiocarcinoma	*FGFR2* N549K, *FGFR2-KCNH7* fusion	3	Derazantinib	8	38.3	PR
1001-37	Cholangiocarcinoma	*FGFR2* C382R	3	Derazantinib	10	32.8	PR
1001-41^b^	HR+/HER2− breast cancer	*FGFR2* rearrangement	13	Palbociclib; SYK/FLT3 inhibitor^d^; PIM kinase inhibitor^d^	12	12.1	PR
2001-50	HR+/HER2− breast cancer	None	6	None	10	25	PR
1001-74^b^	TNBC	None	8	None	12	16.1	PR
1001-64^b^	Prostate adenocarcinoma	*FGFR2* amplification	13	SUMO-activating enzyme inhibitor^d^	12	21.9	PR
1001-79^b^	Cholangiocarcinoma	*FGFR2*-*CCDC6*.F17C2^c^	4	Pemigatinib	12	28	PR
1001-34	Cholangiocarcinoma	*FGFR2*-*AHCYL1*.F17A2	4	Infigratinib	10	33.9	SD
1001-15	HR+/HER2− breast cancer	None	7	NO synthase inhibitor^d^	8	26.1	SD
2001-24	TNBC	None	3	None	5	24.6	SD
1001-66^b^	Rectum adenocarcinoma	*FGFR1* amplification	5	Pemigatinib	12	26	SD
1001-67^b^	Epithelioid mesothelioma	None	3	Bevacizumab	12	50.4	SD
1001-05	Salivary gland adenocarcinoma	None	3	Ceritinib; abemaciclib	5	36.4	SD

Abbreviations: DRDE, dose recommended for dose expansion; FGFR, fibroblast growth factor receptor; FLT3, FMS-like tyrosine kinase 3; HER2−, human epidermal growth factor 2-negative; HR+, hormone receptor-positive; NO, nitric oxide; PIM, proviral integration site of Moloney murine leukemia virus; PR, partial response; RECIST, Response Evaluation Criteria in Solid Tumors; SD, stable disease; SUMO, small ubiquitin-like modifier; SYK, spleen tyrosine kinase; TNBC, triple-negative breast cancer.

^a^Indicates historical *FGFR* mutational status obtained from medical records review.

^b^Indicates patient treated at DRDE.

^c^Indicates *FGFR* mutational status obtained from next-generation sequencing performed during study screening and prior to study entry.

^d^Investigational agent under study.

Among the 11 efficacy-evaluable patients with cholangiocarcinoma, PRs were noted in 3 patients and SD lasting ≥24 weeks was observed in one other. All 4 patients had *FGFR2* alterations [*FGFR2* fusions (*n* = 2), *FGFR2* fusion and *FGFR2* mutation (*n* = 1), and *FGFR2* mutation (*n* = 1)] documented either historically from medical records (*n* = 3) or prospectively by next-generation sequencing at baseline (*n* = 1; see Table 3 for details). All 4 patients had been previously treated with one or more FGFR tyrosine kinase inhibitor. Further PRs were noted in 2 of 3 efficacy-evaluable patients with HR-positive/HER2-negative breast cancer, with SD lasting ≥24 weeks in one other; PRs were noted in 1 of 5 patients with TNBC and one patient with CRPC; an additional SD lasting ≥24 weeks was observed in a patient with TNBC.

### Pharmacokinetics

**Figure 2.**
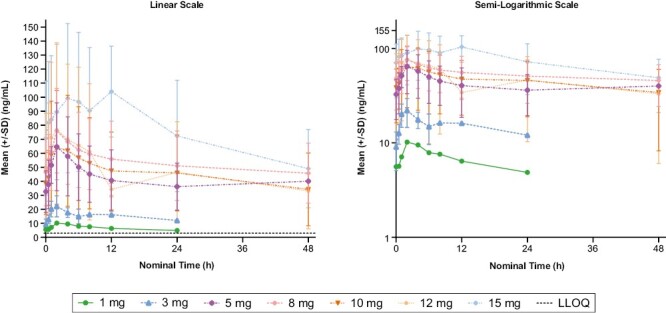
Mean (±SD) plasma concentration-time plots of multiple-dose tinengotinib on cycle 1 day 28 by dose (dose-escalation phase). In lower-dose cohorts (1-5 mg), the multiple-dose plasma concentrations were measured over 24 hours; in other dose cohorts, concentrations were measured over 48 hours. Abbreviations: LLOQ, lower limit of quantification; SD, standard deviation.

Preliminary clinical pharmacokinetics were analyzed in 40 evaluable patients who received at least one dose of tinengotinib and had at least one post-baseline pharmacokinetic sample collected (Supplementary Table S5). Mean tinengotinib plasma concentrations (linear and semi-logarithmic forms) on day 28 of cycle 1 are shown in Fig. 2. Following oral administration, T_max_ was attained after ~2-5 hours on average. Mean t_1/2_ was measurable for doses of 8 mg and above and ranged between 28 and 34 hours. Consistent with the observed t_1/2_ and daily dosing regimen, mean accumulation ratios for AUC_0-24_ ranged from 2.5 to 2.8 for all doses. Exposure was observed to increase in a less-than-proportional manner, and mean AUC_0-24_ values for both single doses and at steady state for the 15 mg dose were approximately twice those observed for the 5 mg dose (1.98 and 1.91, respectively).

### Exploratory Biomarker and Pharmacodynamic Analyses

Prospective biomarker data were available for one patient with a PR who had cholangiocarcinoma harboring an *FGFR2* fusion. Between baseline and cycle 3 day 1, the patient had a 4.8-fold increase in plasma FGF-23 (a marker of FGFR inhibition) levels, which was accompanied by 30% reduction in target tumor size (Supplementary Fig. S3). Additionally, tinengotinib displayed robust antitumor activity and achieved 80% TGI in the CC6204 PDX *FGFR2-BICC1* gene fusion model (Supplementary Fig. S4), suggesting its potential clinical application in patients with *FGFR2* fusion-positive cholangiocarcinoma.

The median inhibitory concentration (IC_50_) for pemigatinib, the positive control in the KATO III cell line model, was 4.4 nM, consistent with published data.^11^ As shown in Supplementary Table S6, the IC_50_, IC_70_, and IC_85_ values for tinengotinib were 16.5 nM (6.51 ng/mL), 37.85 nM (14.95 ng/mL), and 90.28 nM (35.65 ng/mL), respectively, in the same assay. The steady-state C_min_ of 5 mg of tinengotinib was 32.7 ng/mL and the steady-state C_min_ of 12 mg/day of tinengotinib was 46.0 ng/mL as shown in Supplementary Table S5.

## Discussion

This first-in-human phase I study evaluated tinengotinib, a novel multi-target tyrosine kinase inhibitor targeting FGFR 1-3, JAK 1 and 2, VEGFRs, and Aurora A and B, in patients with advanced solid tumors. The DRDE was established at 12 mg orally once daily on a 28-day cycle. The most common drug-related adverse events were vascular (hypertension), gastrointestinal (diarrhea, stomatitis, nausea, vomiting), and dermatologic (palmar-plantar erythrodysesthesia syndrome), a safety profile that is consistent with other multi-kinase inhibitors that target FGFRs and VEGFRs.^12,13^ Most drug-related adverse events associated with tinengotinib were mild-to-moderate in severity, and manageable with dose modifications or standard therapies.

Hypertension, a well-documented on-target effect of VEGF inhibition,^14^ was the most frequently reported adverse event with tinengotinib. An exposure-response model suggested that the risk of grade ≥3 hypertension was uniform and consistent across all doses (Supplementary Fig. S2). Treatment-emergent hypertension was effectively managed with dose modifications and/or antihypertensive agents in this study; however, a more proactive approach has been adopted in ongoing and future clinical studies to minimize unnecessary dose interruptions. This includes the exclusion of patients with uncontrolled hypertension at screening (defined as systolic blood pressure ≥150 mmHg and/or diastolic blood pressure ≥90 mmHg), and detailed guidelines for the management of treatment-emergent hypertension (ie, daily monitoring and reporting of blood pressure, and guidelines for tinengotinib dose modifications and the use of antihypertensive agents by CTCAE grade).

To date, a number of Aurora kinase inhibitors have been evaluated in clinical trials.^15^ The most commonly observed adverse events in those trials were febrile neutropenia and stomatitis/mucosal inflammation, which were considered to be mechanism-based toxicities for this class of inhibitors.^15,16^ As a potent pan-Aurora inhibitor, tinengotinib resulted in myelosuppression including a reduction in neutrophils, monocytes, and lymphocytes in preclinical toxicity studies (in-house source data). Interestingly, although stomatitis was reported in 9 (28.1%) of the patients in the phase II study (NCT04919642)^17^ and 14 (29.2%) in this study, hematologic toxicity was uncommon in both studies. This might be due to reduced bone marrow distribution of the drug in human subjects, but further investigation is needed to determine the reason for this observation.

Preclinical pharmacokinetic/pharmacodynamic (PK/PD) models indicated that the lowest active dose of tinengotinib was 5 mg/kg, which resulted in 61.5% TGI and an AUC of 707 h·ng/mL.^3^ In the present study, steady-state exposure (AUC) at a dose of 5 mg/day was 1053 h·ng/mL, and the exposure of AUC at a dose of 12 mg/day was 1374.4 h·ng/mL, which exceeded the minimal efficacious AUC of 707 h·ng/mL defined in preclinical PK/PD models.^3^ The IC_70_ and IC_85_ values and C_min_ at 5 mg/day and 12 mg/day suggest that sufficient FGFR2 inhibition (>70%) can be maintained if tinengotinib is dosed at ≥5 mg once daily or FGFR2 inhibition >85% can be maintained if tinengotinib is dosed at ≥12 mg once daily. This dose level was also accompanied by disease control in some patients, suggesting that the minimum dose associated with clinical benefit is 5 mg/day. Although exposure to tinengotinib increased in a less-than-proportional manner with dose, the efficacious range could be defined from 5 mg/day to 12 mg/day. Two ongoing trials of tinengotinib (Clinicaltrials.gov NCT04742959 and NCT04919642) include a pharmacokinetic run-in study, intensive pharmacokinetic sample collection to determine how the body metabolizes tinengotinib, and additional sample collection to generate pooled pharmacokinetic parameters of tinengotinib in a larger population.

Tinengotinib showed encouraging preliminary antitumor activity in heavily pretreated patients with a variety of advanced solid tumors including cholangiocarcinoma, HR-positive/HER2-negative breast cancer, TNBC, and CRPC. A notable observation was the strong efficacy signal with tinengotinib in patients with cholangiocarcinoma despite those patients having had 3-5 prior lines of standard therapy. All 4 patients who achieved a PR or SD ≥24 weeks with tinengotinib had *FGFR2* genomic alterations (rearrangements, fusions, or mutations) at baseline and had been previously treated with an FGFR inhibitor. It has been reported that a spectrum of *FGFR* alterations occurs in cholangiocarcinoma through amplification, activating mutations, or fusions via gene rearrangements.^18,19^ The development of FGFR inhibitors has led to approvals of pemigatinib, infigratinib, and futibatinib for patients with *FGFR2* fusion or rearranged cholangiocarcinoma after failure of first-line chemotherapy based on objective response rates of 36%, 23%, and 42%, respectively.^20-22^ Although FGFR inhibitors have clinical efficacy in cholangiocarcinoma harboring *FGFR2* fusions, many patients ultimately develop disease progression after 7-9 months^20,23^ as a result of secondary mutations in the *FGFR2* kinase domain. Treatment of FGFR inhibitor-refractory cholangiocarcinoma represents an area of unmet need.^19,24^ It is notable that tinengotinib, as a multi-target tyrosine kinase inhibitor, demonstrated inhibitory activity against FGFR kinases in vitro^3^ as well as in patients harboring *FGFR2* kinase domain (N549K) or transmembrane domain (C382R) mutations in the present study. Supportive biomarker data were also documented in one patient with cholangiocarcinoma after tinengotinib therapy that showed a 4.8-fold increase in FGF-23 (a marker of FGFR inhibition) plasma levels accompanied by 30% reduction in target tumor size at cycle 3 day 1. Based on these encouraging findings, tinengotinib has received a fast-track designation from the US Food and Drug Administration for further development in cholangiocarcinoma. A phase II study is underway to better understand the underlying mechanisms of response and resistance to tinengotinib in patients with *FGFR*-altered or *FGFR*-wild-type cholangiocarcinoma (Clinicaltrials.gov, NCT04919642). That study will address the limitations of this study, in which biomarker data regarding *FGFR* status were collected retrospectively from historical records in most patients, and includes biomarker profiling at baseline, during treatment, and upon response/progression. That study will investigate tinengotinib monotherapy in patients with cholangiocarcinoma with *FGFR2* fusions for whom prior FGFR inhibitor therapy was unsuccessful, or those who responded to previous FGFR inhibitor(s), or with other *FGFR* alterations, or wild-type *FGFR*.

The efficacy signal in patients with TNBC is also encouraging and consistent with preclinical studies that identified tinengotinib as a potential candidate for this type of tumor.^3^ TNBC encompasses multiple subtypes characterized by distinct genetic drivers, including Aurora A and B kinase in the basal-like 1 subtype, and FGFR, PDGFR, and VEGF signaling pathways in the mesenchymal and mesenchymal stem-like subtypes.^25^ In a preclinical study, exposure to tinengotinib inhibited proliferation across all subtypes of TNBC in vitro and in vivo, but did not inhibit breast cancer luminal cell lines in vitro.^3^ Inhibition of Aurora A and B kinase activity was identified as the predominant mechanism of action of tinengotinib in TNBC, with inhibition of other pathways (VEGFR, JAK-STAT, CSF1R, and epidermal growth factor receptor) further contributing to its activity.^3^ FGF/FGFR signaling also plays a pivotal role in prostate cancer and supports FGF- or FGFR-targeted therapeutic strategies for the treatment of prostate cancer.^26^ Based on the PR observed in a patient with CRPC in this study, a phase Ib/II study of tinengotinib, which includes patients with TNBC and CRPC, has recently been initiated (ClinicalTrials.gov: NCT04742959).

The broad spectrum of activity of tinengotinib, in particular, dual inhibition of JAK-STAT and FGFR signaling and blockade of lineage plasticity, may be a factor in the responders as efficacy assessments observed in patients with HR-positive/HER2-negative breast cancer, TNBC, and metastatic CRPC (mCRPC), among others, who did not have *FGFR* alterations at study entry. As previously reported, dual inhibition of FGFR and JAK might benefit patients with mCRPC via lineage reprogramming.^27^ Detailed molecular studies aiming to illustrate pharmacological effects subsequent to JAK-STAT/FGFR inhibition are being conducted in models of mCRPC, and a clinical proof-of-concept study of tinengotinib plus hormonal therapies to treat mCRPC is being planned. Although the underlying molecular mechanism for tinengotinib activity in patients with breast cancer is unclear, there is growing recognition that lineage plasticity plays a critical role in relapse and drug resistance.^28,29^ In TNBC, tumors that are induced to acquire vulnerability by Aurora inhibition may be subsequently targeted and killed by inhibition of additional signal pathways. Thus, targeting a number of cancer hallmarks including proliferation, angiogenesis, epithelial-mesenchymal transition, and immune oncology through the inhibition of several prominent oncogenic pathways may be mechanisms for the activity of tinengotinib in patients with *FGFR*-unaltered breast and prostate cancers. Further studies are needed to confirm these hypotheses.

Some limitations of the present study warrant consideration. These include the limited collection of PK/PD analyses. The ongoing phase Ib/II trial includes one arm specifically designed to evaluate the pharmacokinetics of tinengotinib at a range of doses and using several dosing schedules, with a pharmacokinetics run-in period. Another limitation is the fact that correlative biomarker studies of markers associated with response were not conducted because of the explorative nature of the study. Biomarker analysis of patients with non-FGFR alterations would be of particular interest and may further shed light on other mechanisms involved in response to tinengotinib in our patient population. The phase Ib/II study will also evaluate patient biomarker status, including *FGFR* mutation status.

## Conclusion

In conclusion, tinengotinib was safe and well-tolerated. Based on the totality of safety, efficacy, and PK/PD modeling, the DRDE of 12 mg was established, which exceeded threshold pharmacokinetic levels for efficacy. The preliminary responses observed in patients with cholangiocarcinoma harboring *FGFR2* fusions and/or mutations, HER2-negative breast cancer including TNBC, and CRPC form the basis for further investigations of tinengotinib. Phase II trials have been initiated to evaluate the safety and efficacy of tinengotinib as a single agent or as a combination, and to explore biomarkers for response.

## Supplementary Material

Supplementary material is available at *The Oncologist* online.

oyad338_suppl_Supplementary_Material

## Data Availability

Anonymized individual participant data that underlie the results reported in this article (text, tables, figures, and appendices) and supporting documents (study protocol) may be available upon request beginning 9 months and ending 36 months following article publication. Only requests that have a methodologically sound proposal will be considered. Proposals should be directed to fan_jean@transtherabio.com.
