# Supplementary material for: First-In-Human Phase I Study of Tinengotinib (TT-00420), a Multiple Kinase Inhibitor, as a Single Agent in Patients With Advanced Solid Tumors
Source: Oncologist. 2024 Jan 31;29(4):e514–25. doi: 10.1093/oncolo/oyad338 (PMC10994248; doi:10.1093/oncolo/oyad338)
Supplement: oyad338_suppl_Supplementary_Material [file oyad338_suppl_supplementary_material.docx]

**SUPPLEMENTARY MATERIAL**

# Supplementary methods

## Eligibility criteria

### Inclusion criteria

1. Aged 18–75 years at the time of provision of informed consent.

2. **Dose-escalation cohorts**: Histopathological or cytologically documented locally advanced or metastatic solid tumors who have no available standard therapeutic treatment options.

**Dose-expansion cohort:** Histopathological or cytologically documented cholangiocarcinoma, and locally advanced or metastatic solid tumors (HER2-negative breast cancer or advanced solid tumors).

Preferred indications include:

a. HER2-negative breast cancer as per American Society of Clinical Oncology/College of American Pathologists guidelines:

i. Triple-negative breast cancer who have failed at least one line of prior systemic treatment; or

ii. Hormone receptor-positive/HER2-negative breast cancer who have failed at least one line of prior therapy with a cyclin-dependent kinase (CDK)4/6 inhibitor.

b. Histologically or cytologically diagnosed cholangiocarcinoma (intrahepatic or extrahepatic), meeting any of following baseline requirements:

i. Confirmation of an *FGFR2* fusion.

ii. Confirmation of an *FGFR2* fusion with documented disease progression after at least 1 line of prior fibroblast growth factor receptor (FGFR) inhibitor therapy.

iii. Confirmation of an *FGFR2* rearrangement with documented disease progression after at least 1 line of prior FGFR inhibitor therapy.

c. Diagnosis of a selected indication, including urothelial carcinoma with *FGFR* alterations, gallbladder cancer, gastric cancer, and small cell lung cancer, etc., and have failed at least one line of systemic therapy. Other tumor types may be eligible based on sponsor approval.

3. At least one measurable lesion as defined by Response Evaluation Criteria in Solid Tumors (RECIST) V1.1 criteria for solid tumors.

4. Eastern Cooperative Oncology Group (ECOG) performance status of 0 or 1.

5. Adequate organ function confirmed at Screening and within 10 days of initiating treatment, as evidenced by:

• Absolute neutrophil count (ANC) ≥1.5 × 10^9^/L.

• Hemoglobin ≥9 g/dl.

• Platelets ≥100 × 10^9^/L. (For patients in dose-expansion cohorts, platelets ≥75 × 10^9^/L).

• Aspartate aminotransferase/serum glutamic oxaloacetic transaminase and alanine aminotransferase/serum glutamic pyruvic transaminase ≤2.5 × upper limit of normal (ULN) or ≤5.0 × ULN if liver metastases are present.

• Total bilirubin ≤1.5 × ULN, or direct bilirubin <ULN for patients with total bilirubin levels >1.5 ULN

(for patients in dose-expansion cohorts: total bilirubin ≤3.0 × ULN).

• Serum creatinine ≤1.5 × ULN or calculated 24-hour clearance ≥50 mL/min (by Cockcroft–Gault formula).

• Negative pregnancy test within 72 hours before starting study treatment in all pre-menopausal women and women <12 months after the onset of menopause.

6. Must agree to take sufficient contraceptive methods to avoid pregnancy during the study and until at least 6 months after ceasing study treatment.

7. Able to sign informed consent and to comply with the protocol.

### Exclusion criteria

1. Women who are pregnant or lactating.

2. Women of child-bearing potential who does not use adequate birth control.

3. Patients with any hematologic malignancy, including but not limited to leukemia (any form), lymphoma, and multiple myeloma.

4. Patients with:

a. A history of primary central nervous system tumors or

b. Carcinomatous meningitis.

Note: Patients with treated brain metastases that are off corticosteroid and have been clinically stable for 28 days are eligible for enrollment.

5. Patients with the following mood disorders as judged by the Investigator or a psychiatrist, or as result of patient’s mood assessment questionnaire:

• Medically documented history of or active major depressive episode, bipolar disorder (I or II), obsessive-compulsive disorder, schizophrenia; a history of suicidal attempt or ideation, or homicidal ideation (immediate risk of doing harm to others).

• Common Terminology Criteria for Adverse Events (CTCAE) grade ≥3 anxiety.

• Note that a qualified psychiatrist may overrule the mood assessment questionnaire result, and the patient may be eligible if Investigator and Sponsor agree.

6. Impaired cardiac function or clinically significant cardiac diseases, including but not limited to any of the following:

a. Left ventricular ejection fraction <45% as determined by multigated acquisition scan or echocardiogram.

b. Congenital long QT syndrome.

c. Fridericia-corrected QT interval ≥450 msec on screening electrocardiogram.

d. Unstable angina pectoris ≤3 months prior to starting study drug.

e. Acute myocardial infarction ≤3 months prior to starting study drug.

7. Patients with:

a. Unresolved diarrhea CTCAE grade ≥2, or

b. Impairment of gastrointestinal (GI) function, or

c. GI disease that may significantly alter the absorption of TT-00420 (e.g., ulcerative diseases, uncontrolled nausea, vomiting, diarrhea, malabsorption syndrome, or small bowel resection).

8. Other concurrent severe and/or uncontrolled concomitant medical conditions (e.g., uncontrolled hypertriglyceridemia [triglycerides >500 mg/dL], active or uncontrolled infection) that could cause unacceptable safety risks or compromise compliance with the protocol.

9. Patients who have received chemotherapy, targeted therapy, or immunotherapy ≤4 weeks (6 weeks for nitrosourea or mitomycin-C) prior to starting study drug or who have not recovered from side effects of such therapy.

10. Patients who have received wide-field radiotherapy ≤4 weeks or limited-field radiation for palliation ≤2 weeks prior to starting study drug or who have not recovered from side effects of such therapy.

11. Patients who have undergone major surgery ≤4 weeks prior to starting study drug or who have not recovered from side effects of such therapy.

12. Patients who have been treated with any hematopoietic colony-stimulating growth factors (e.g., granulocyte colony-stimulating factor, granulocyte–macrophage colony-stimulating factor) ≤4 weeks prior to starting study drug.

13. Patients who are currently receiving treatment with therapeutic doses of warfarin sodium (Coumadin^®^) or any other coumarin-derivative anticoagulants.

14. Patients who have received systemic corticosteroids ≤2 weeks prior to starting study drug or who have not recovered from the side effects of such treatment. Note: Patients that are taking hormone replacement doses of steroids are eligible.

15. Patients who are currently receiving treatment with medication that has known risk to prolong the QT interval or induce torsade de pointes, and the treatment cannot either be discontinued or switched to a different medication prior to starting study drug.

16. Patients who are receiving high to moderate cytochrome P450 (CYP) 3A inhibitors and inducers.

17. Known diagnosis of human immunodeficiency virus (HIV) infection (HIV testing is not mandatory; patients with well-controlled HIV may be enrolled per investigator’s discretion and Sponsor approval).

18. Known history of active infection with hepatitis B (e.g., hepatitis B surface antigen reactive) or hepatitis C (e.g., HCV RNA [qualitative] is detected).

19. Has received a live-virus vaccination within 30 days of planned first dose. Note: Seasonal influenza vaccines are permitted.

20. Inability to swallow or tolerate oral medication.

21. Has a history or current evidence of any condition, therapy, or laboratory abnormality that, in the opinion of the Investigator, might confound the results of the trial, interfere with the patient’s safe participation and compliance in the trial.

**Supplementary Table S1.** Dose-limiting toxicity definitions

| **Toxicity** | **DLT Criteria** |
| --- | --- |
| Hematologic | CTCAE grade ≥3 neutropenia lasting >5 consecutive days |
|  | CTCAE grade 3, grade 4 thrombocytopenia with clinically significant hemorrhage |
|  | CTCAE grade ≥3 clinically significant lymphopenia lasting >7 consecutive days |
|  | CTCAE grade ≥3 anemia if judged to be a hemolytic process due to tinengotinib |
|  | Febrile neutropenia (ANC <1.0 x 10^9^/L, fever ≥38.5°C) |
| Gastrointestinal | CTCAE grade 3 vomiting, nausea, and diarrhea lasting >72 hours despite adequate supportive care |
|  | CTCAE grade 4 nausea, vomiting and diarrhea of any duration |
| Mood alteration | CTCAE grade 2 mood alteration NOT resolved to grade ≤1 within 14 days despite medical treatment |
|  | CTCAE grade ≥3 mood alteration |
| Neurotoxicity | CTCAE grade ≥3 |
| Cardiac | QTcF change from baseline >60 msec (but absolute QTcF ≤500 msec) |
|  | Grade ≥3 (QTcF >500 msec) prolonged QTcF interval |
|  | CTCAE grade ≥3 cardiac event/disease that is symptomatic or requires medical intervention |
| Hepatic | CTCAE grade 3 total bilirubin for >7 consecutive days |
|  | CTCAE grade 4 total bilirubin |
|  | CTCAE grade 3 AST or ALT for >5 consecutive days |
|  | CTCAE grade 4 AST or ALT |
|  | Hy’s Law: AST/ALT ≥3 x ULN with total bilirubin ≥2 x ULN in the absence of a cause other than study drug |
| Renal | CTCAE grade 3 serum creatinine for >7 consecutive days |
|  | CTCAE grade 4 serum creatinine |
|  | CTCAE grade ≥2 hematuria and [+3] proteinuria >7 consecutive days |
| Dermatologic | CTCAE grade ≥2 phototoxicity |
|  | Any grade skin toxicity (i.e., rash) resulting in interruption of tinengotinib for >21 consecutive days |
| Endocrine | CTCAE grade 2 hyperglycemia (confirmed with a repeat fasting plasma glucose within 24 hours) that does not resolve to grade 0 within 14 consecutive days (after initiation of glimepiride, metformin or glibenclamide) |
|  | CTCAE grade ≥3 hyperglycemia (confirmed with a repeat fasting plasma glucose within 24 hours) |
| Metabolic/Laboratory | CTCAE grade 3 symptomatic amylase and/or lipase, not reversible to CTCAE grade ≤2 for >7 consecutive days |
|  | CTCAE grade 4 symptomatic amylase and/or lipase |
| Pancreatitis | CTCAE grade ≥2 |
| General | Any grade adverse event leading to dose interruption >7 consecutive days despite medical treatment |
|  | 2nd occurrence of an initial non-DLT toxicity leading to a dose reduction within the 1st cycle |
|  | Any other CTCAE grade ≥3 adverse event not listed above that might be considered as a DLT per Investigators’ evaluation and discretion |

Abbreviations: ALT, alanine aminotransferase; ANC, absolute neutrophil count; AST, aspartate aminotransferase; CTCAE, Common Terminology Criteria for Adverse Events; DLT, dose-limiting toxicity; QTcF, Fridericia-corrected QT interval; ULN, upper limit of normal.

**Supplementary Table S2.** Patient baseline demographics and clinical characteristics by dose level (full analysis set)

|  | **Dose**  **Escalation** | | | | | | | | **Dose Expansion** | **Total** |
| --- | --- | --- | --- | --- | --- | --- | --- | --- | --- | --- |
|  | **1 mg (*n*=1)** | **3 mg (*n*=1)** | **5 mg (*n*=4)** | **8 mg (*n*=10)** | **10 mg (*n*=6)** | **12 mg (*n*=12)** | **15 mg (*n*=6)** | **Total (*n*=40)** | **12 mg**  **(*n*=8)** | **(*n*=48)** |
| Age (years), years | | | | | | | | | | |
| Median (range) | 75.1 (NA) | 42.4 (NA) | 53.3  (32–62) | 56.1  (32–64) | 53.3  (28–79) | 66.6  (49–74) | 53.8  (43–65) | 57.4  (28–79) | 44.6  (25–73) | 56.8  (25–79) |
| <60, *n* (%) | 0 | 1 (100) | 2 (50.0) | 7 (70.0) | 5 (83.3) | 3 (25.0) | 5 (83.3) | 23 (57.5) | 6 (75.0) | 29 (60.4) |
| ≥60, *n* (%) | 1 (100) | 0 | 2 (50.0) | 3 (30.0) | 1 (16.7) | 9 (75.0) | 1 (16.7) | 17 (42.5) | 2 (25.0) | 19 (39.6) |
| Sex, *n* (%) | | | | | | | | | | |
| Male | 1 (100) | 1 (100) | 2 (50.0) | 4 (40.0) | 2 (33.3) | 5 (41.7) | 1 (16.7) | 16 (40.0) | 6 (75.0) | 22 (45.8) |
| Female | 0 | 0 | 2 (50.0) | 6 (60.0) | 4 (66.7) | 7 (58.3) | 5 (83.3) | 24 (60.0) | 2 (25.0) | 26 (54.2) |
| Race, *n* (%) | | | | | | | | | | |
| White | 1 (100) | 1 (100) | 1 (25.0) | 5 (50.0) | 4 (66.7) | 8 (66.7) | 6 (100) | 26 (65.0) | 7 (87.5) | 33 (68.8) |
| Black or African American | 0 | 0 | 2 (50.0) | 1 (10.0) | 0 | 3 (25.0) | 0 | 6 (15.0) | 0 | 6 (12.5) |
| Asian | 0 | 0 | 1 (25.0) | 3 (30.0) | 2 (33.3) | 0 | 0 | 6 (15.0) | 0 | 6 (12.5) |
| Other | 0 | 0 | 0 | 1 (10.0) | 0 | 1 (8.3) | 0 | 2 (5.0) | 1 (12.5) | 3 (6.3) |
| ECOG performance status, *n* (%) | | | | | | | | | | |
| 0 | 0 | 0 | 3 (75.0) | 2 (20.0) | 1 (16.7) | 0 | 2 (33.3) | 8 (20.0) | 1 (12.5) | 9 (18.8) |
| 1 | 1 (100) | 1 (100) | 1 (25.0) | 8 (80.0) | 5 (83.3) | 12 (100) | 4 (66.7) | 32 (80.0) | 7 (87.5) | 39 (81.3) |
| Primary tumor site, *n* (%) | | | | | | | | | | |
| Liver | 0 | 0 | 0 | 1 (10.0) | 2 (33.3) | 2 (16.7) | 2 (33.3) | 7 (17.5) | 3 (37.5) | 10 (20.8) |
| Breast | 0 | 0 | 1 (25.0) | 4 (40.0) | 2 (33.3) | 1 (8.3) | 0 | 8 (20.0) | 1 (12.5) | 9 (18.8) |
| Colon | 0 | 0 | 0 | 0 | 0 | 1 (8.3) | 0 | 1 (2.5) | 1 (12.5) | 2 (4.2) |
| Lung | 1 (100) | 0 | 0 | 1 (10.0) | 0 | 0 | 0 | 2 (5.0) | 0 | 2 (4.2) |
| Esophagus | 0 | 0 | 0 | 1 (10.0) | 0 | 0 | 1 (16.7) | 2 (5.0) | 0 | 2 (4.2) |
| Ovary | 0 | 0 | 0 | 0 | 0 | 0 | 2 (33.3) | 2 (5.0) | 0 | 2 (4.2) |
| Salivary gland | 0 | 1 (100) | 1 (25.0) | 0 | 0 | 0 | 0 | 2 (5.0) | 0 | 2 (4.2) |
| Soft tissue sarcoma | 0 | 0 | 1 (25.0) | 0 | 1 (16.7) | 0 | 0 | 2 (5.0) | 0 | 2 (4.2) |
| Other^a^ | 0 | 0 | 1 (25.0) | 3 (30.0) | 1 (16.7) | 8 (66.7) | 1 (16.7) | 14 (35.0) | 3 (37.5) | 17 (35.4) |
| Prior therapies, *n* (%) | 1 (100) | 1 (100) | 4 (100) | 10 (100) | 6 (100) | 12 (100) | 6 (100) | 40 (100) | 8 (100) | 48 (100) |
| Radiation | 1 (100) | 1 (100) | 3 (75.0) | 5 (50.0) | 3 (50.0) | 6 (50.0) | 3 (50.0) | 2 (55.0) | 2 (25.0) | 24 (50.0) |
| Surgery | 1 (100) | 1 (100) | 4 (100) | 10 (100) | 6 (100) | 12 (100) | 6 (100) | 40 (100) | 8 (100) | 48 (100) |
| Anticancer medication | 1 (100) | 1 (100) | 3 (75.0) | 10 (100) | 6 (100) | 12 (100) | 6 (100) | 39 (97.5) | 8 (100) | 47 (97.9) |
| Chemotherapy | 1 (100) | 1 (100) | 2 (50.0) | 10 (100) | 6 (100) | 12 (100) | 5 (83.3) | 37 (92.5) | 8 (100) | 45 (93.8) |
| Hormonal therapy | 0 | 0 | 0 | 1 (10.0) | 1 (16.7) | 3 (25.0) | 0 | 5 (12.5) | 1 (12.5) | 6 (12.5) |
| Immunotherapy | 1 (100) | 1 (100) | 1 (25.0) | 8 (80.0) | 0 | 3 (25.0) | 3 (50.0) | 17 (42.5) | 1 (12.5) | 18 (37.5) |
| Targeted therapy | 0 | 0 | 2 (50.0) | 4 (40.0) | 2 (33.3) | 3 (25.0) | 2 (33.3) | 13 (32.5) | 2 (25.0) | 15 (31.3) |
| Other | 0 | 0 | 1 (25.0) | 0 | 0 | 1 (8.3) | 0 | 2 (5.0) | 0 | 2 (4.2) |
| Lines of therapy, *n* (%) | | | | | | | | | | |
| 0 | 0 | 0 | 1 (25.0) | 0 | 0 | 0 | 0 | 1 (2.5) | 0 | 1 (2.1) |
| 1 | 0 | 1 (100) | 0 | 2 (20.0) | 1 (16.7) | 0 | 2 (33.3) | 6 (15.0) | 4 (50.0) | 10 (20.8) |
| 2 | 0 | 0 | 0 | 1 (10.0) | 0 | 1 (8.3) | 0 | 2 (5.0) | 1 (12.5) | 3 (6.3) |
| 3 | 1 (100) | 0 | 2 (50.0) | 3 (30.0) | 2 (33.3) | 4 (33.3) | 1 (16.7) | 13 (32.5) | 0 | 13 (27.1) |
| ≥4 | 0 | 0 | 1 (25.0) | 4 (40.0) | 3 (50.0) | 7 (58.3) | 3 (50.0) | 18 (45.0) | 3 (37.5) | 21 (43.8) |

Abbreviations: ECOG, Eastern Cooperative Oncology Group; HNSCC, head and neck squamous cell carcinoma; NA, not applicable; TNBC, triple-negative breast cancer.

^a^Bone sarcoma (*n*=1), gall bladder (*n*=1), gall bladder ducts (*n*=1), head and neck (*n*=1), oral cavity (*n*=1), pancreas (*n*=1), peritoneum (*n*=1), prostate (*n*=1), rectum (*n*=1), testis (*n*=1), uterus (*n*=1), other (*n*=6).

**Supplementary Table S3.** Summary of dose-limiting toxicities (dose-determining set)

|  | **Dose Escalation** | | | | | | | |  |
| --- | --- | --- | --- | --- | --- | --- | --- | --- | --- |
| **System Organ Class Preferred Term** | **1 mg (*n*=1)** | **3 mg (*n*=1)** | **5 mg (*n*=4)** | **8 mg (*n*=9)** | **10 mg (*n*=3)** | **12 mg (*n*=9)** | **15 mg (*n*=5)** | **Total (*n*=32)** |  |
| Subjects who had a dose-limiting toxicity | 0 | 0 | 0 | 1 (11.1) | 0 | 0 | 2 (40.0) | 3 (9.4) |  |
| Vascular disorders | 0 | 0 | 0 | 0 | 0 | 0 | 2 (40.0) | 2 (6.3) |  |
| Hypertension | 0 | 0 | 0 | 0 | 0 | 0 | 2 (40.0) | 2 (6.3) |  |
| Skin and subcutaneous tissue disorders | 0 | 0 | 0 | 1 (11.1) | 0 | 0 | 0 | 1 (3.1) |  |
| Palmar-plantar erythrodysesthesia syndrome | 0 | 0 | 0 | 1 (11.1) | 0 | 0 | 0 | 1 (3.1) |  |

Data are presented as No. (%).

**Supplementary Table S4.** Overall summary of adverse events (safety set)

| **Patients with event, *n* (%)** | **Dose escalation** | | | | | | | | **Dose expansion** | **Combined 12 mg (*n*=20)** | **Total (*n*=48)** |
| --- | --- | --- | --- | --- | --- | --- | --- | --- | --- | --- | --- |
|  | **1 mg (*n*=1)** | **3 mg (*n*=1)** | **5 mg (*n*=4)** | **8 mg (*n*=10)** | **10 mg (*n*=6)** | **12 mg (*n*=12)** | **15 mg (*n*=6)** | **Total (*n*=40)** | **12 mg (*n*=8)** |  |  |
| AE | 1 (100) | 1 (100) | 4 (100) | 9 (90.0) | 6 (100) | 12 (100) | 6 (100) | 39 (97.5) | 8 (100) | 20 (100) | 47 (97.9) |
| TEAE | 1 (100) | 1 (100) | 4 (100) | 9 (90.0) | 6 (100) | 12 (100) | 6 (100) | 39 (97.5) | 8 (100) | 20 (100) | 47 (97.9) |
| Study drug-related^a^ TEAE | 0 | 0 | 3 (75.0) | 8 (80.0) | 6 (100) | 11 (91.7) | 5 (83.3) | 33 (82.5) | 8 (100) | 19 (95.0) | 41 (85.4) |
| grade ≥3 TEAE | 0 | 0 | 1 (25.0) | 6 (60.0) | 5 (83.3) | 9 (75.0) | 5 (83.3) | 26 (65.0) | 5 (62.5) | 14 (70.0) | 31 (64.6) |
| Study drug-related^a^ grade ≥3 TEAE | 0 | 0 | 1 (25.0) | 4 (40.0) | 2 (33.3) | 6 (50.0) | 3 (50.0) | 16 (40.0) | 5 (62.5) | 11 (55.0) | 21 (43.8) |
| TEAE leading to dose adjustment/temporary interruption | 0 | 0 | 2 (50.0) | 5 (50.0) | 5 (83.3) | 8 (66.7) | 4 (66.7) | 24 (60.0) | 7 (87.5) | 15 (75.0) | 31 (64.6) |
| Study drug-related^a^ TEAE leading to dose adjustment/temporary interruption | 0 | 0 | 1 (25.0) | 5 (50.0) | 3 (50.0) | 6 (50.0) | 3 (50.0) | 18 (45.0) | 7 (87.5) | 13 (65.0) | 25 (52.1) |
| TEAE leading to study drug permanent discontinuation | 0 | 0 | 0 | 2 (20.0) | 4 (66.7) | 3 (25.0) | 3 (50.0) | 12 (30.0) | 0 | 3 (15.0) | 12 (25.0) |
| Study drug-related^a^ TEAE leading to study drug permanent discontinuation | 0 | 0 | 0 | 1 (10.0) | 4 (66.7) | 1 (8.3) | 0 | 6 (15.0) | 0 | 1 (5.0) | 6 (12.5) |
| Treatment-emergent SAE | 0 | 0 | 0 | 5 (50.0) | 4 (66.7) | 5 (41.7) | 4 (66.7) | 18 (45.0) | 4 (50.0) | 9 (45.0) | 22 (45.8) |
| Study drug-related^a^ treatment-emergent SAE | 0 | 0 | 0 | 3 (30.0) | 1 (16.7) | 0 | 1 (16.7) | 5 (12.5) | 3 (37.5) | 3 (15.0) | 8 (16.7) |
| TEAE leading to death | 0 | 0 | 0 | 0 | 0 | 0 | 0 | 0 | 0 | 0 | 0 |

Abbreviations: AE, adverse event; SAE, serious adverse event; TEAE, treatment-emergent adverse event.

^a^Study drug-related is defined as a suspected relationship between the AE and study drug; a missing causality was regarded as the AE being related to the study drug.

**Supplementary Table S5.** Tinengotinib pharmacokinetic parameters during cycle 1 (pharmacokinetic evaluable set)

| **Tinengotinib dose** |  | **C_max_, ng/mL** | **T_max_, h** | **AUC_0-24_, h*ng/mL** | **Half-life, h** | **C_min_, ng/mL** | **AR-C_max_** | **AR-AUC** |
| --- | --- | --- | --- | --- | --- | --- | --- | --- |
| **Day 1/Cycle 1** |  |  |  |  |  |  |  |  |
| 5 mg (*n*=4) | Mean | 24.6 | 2.1 | 368.1 | – | – | – | – |
|  | CV% | 37.5 | 4.0 | 34.2 |  |  |  |  |
| 8 mg (*n*=10) | Mean | 47.8 | 2.4 | 665.0 | 28.0 | – | – | – |
|  | CV% | 39.8 | 39.9 | 48.7 | 13.3 |  |  |  |
| 10 mg (*n*=6) | Mean | 53.7 | 2.5 | 760.2 | 34.0 | – | – | – |
|  | CV% | 49.2 | 51.2 | 38.2 | 5.7 |  |  |  |
| 12 mg (*n*=12) | Mean | 35.5 | 5.3 | 540.5 | 27.3 | – | – | – |
|  | CV% | 68.8 | 128.2 | 51. 3 | 41.2 |  |  |  |
| 15 mg (*n*=6) | Mean | 51.9 | 3.5 | 730.7 | 33.4 | – | – | – |
|  | CV% | 42. 9 | 118.0 | 32.2 | 27.7 |  |  |  |
|  |  | AUC_0-24_ ratio 5 mg:15 mg: 1.98 | | | | | | |
| **Day 28/Cycle 1 or Day 1/Cycle 2** | |  |  |  |  |  |  |  |
| 5 mg (*n*=4) | Mean | 64.8 | 2.3 | 1053.4 | – | 32.7 | 2.7 | 2.8 |
|  | CV% | 41.7 | 49.1 | 41.2 |  | 39.6 | 11.3 | 13.3 |
| 8 mg (*n*=10) | Mean | 76.6 | 1.8 | 1386.3 | – | 45.6 | 1.9 | 2.6 |
|  | CV% | 39. 2 | 27.5 | 45.3 |  | 53.0 | 30.7 | 34.2 |
| 10 mg (*n*=6) | Mean | 64.6 | 2.7 | 1221.0 | – | 38.4 | 2.0 | 2.5 |
|  | CV% | 51.0 | 34.3 | 49.3 |  | 46.9 | 49.2 | 49.6 |
| 12 mg (*n*=12) | Mean | 79.1 | 3.4 | 1374.4 | – | 46.0 | 2.6 | 2.8 |
|  | CV% | 76.8 | 80.8 | 80.2 |  | 86.9 | 61.0 | 40.2 |
| 15 mg (*n*=6) | Mean | 99.4 | 5.3 | 2015.0 | – | 70.3 | 2.2 | 2.7 |
|  | CV% | 34.7 | 35.1 | 43.1 |  | 48.0 | 55.6 | 36.6 |
|  |  | AUC_0-24_ ratio 5 mg:15 mg: 1.91 | | | | | | |

Abbreviations: AR, accumulation ratio; AUC_0-24_, area under the time-concentration curve from 0 to 24 hours; C_max_, maximum concentration; CV, coefficient of variance; t_max_, time to C_max_.

**Supplementary Table S6.** Fibroblast growth factor receptor 2 phosphorylation was assessed in the KATO III cell line following treatment with tinengotinib. Cells were cultured in healthy donor plasma spiked with serial dilutions of tinengotinib. Receptor phosphorylation was determined by enzyme-linked immunosorbent assay.

|  | **nmol/L** | **ng/mL** |
| --- | --- | --- |
| IC_50_ | 16.5 | 6.51 |
| IC_70_ | 37.85 | 14.95 |
| IC_85_ | 90.28 | 35.65 |
| IC_90_ | 142.00 | 56.07 |

Abbreviation: IC, inhibitory concentration.

**Supplementary Figure S1**. Summary of analysis sets (all enrolled patients)

**
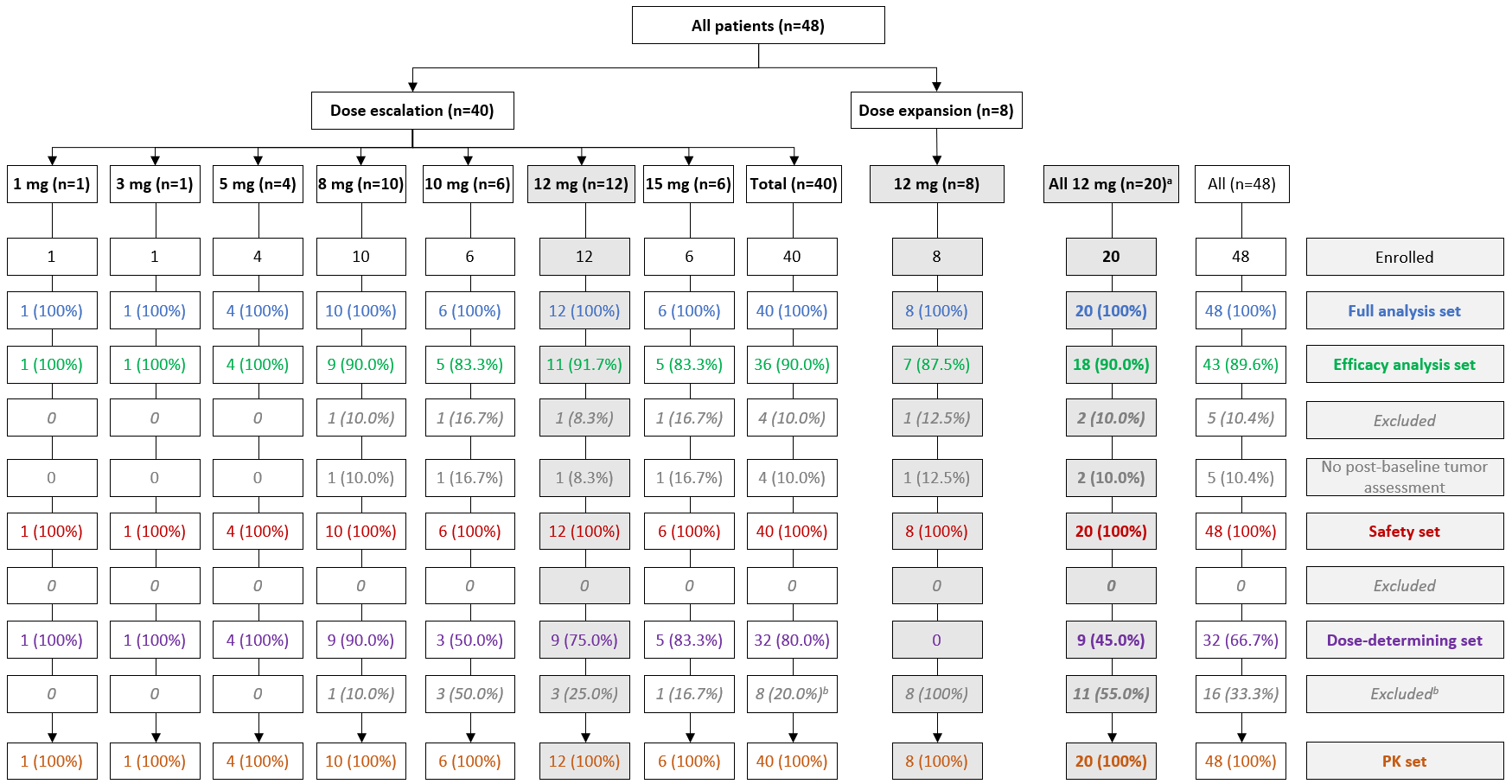
**

^a^Dose-escalation 12 mg and dose-expansion cohorts.

^b^Eight patients did not meet the dose-limiting toxicity criteria. Patients who did not have a dose-limiting toxicity in the first cycle had to have received ≥21 doses of tinengotinib in the first cycle.

**Supplementary Figure S2.** Probability of grade ≥3 hypertension vs tinengotinib plasma concentrations at 2 hours steady-state **(A)** and 24 hours steady-state **(B)** on day 8 of cycle 1

**
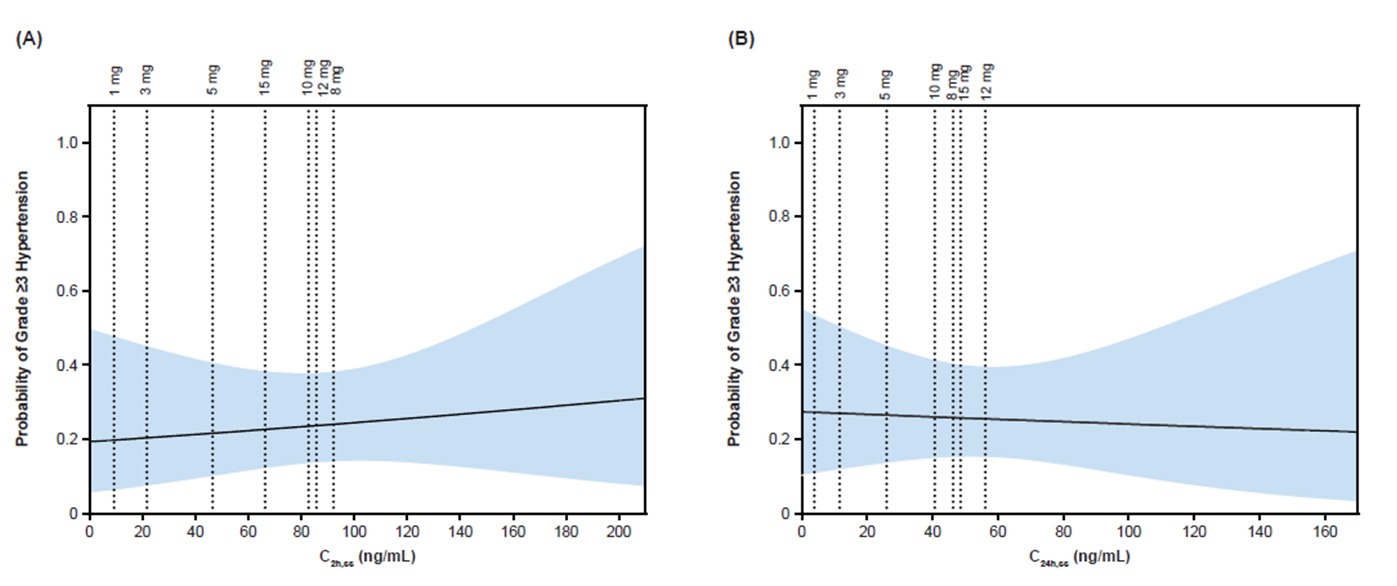
**

Abbreviations: C_2h,ss_ plasma concentrations at 2 hours steady-state; C_24h,ss_ plasma concentrations at 24 hours steady-state.

**Supplementary Figure S3.** Plasma FGF-23 levels **(A)** and changes in target tumor size **(B)** at baseline and during treatment in a patient with cholangiocarcinoma harboring an *FGFR2* fusion who had a partial response (unconfirmed) with tinengotinib.

**(A)**

**
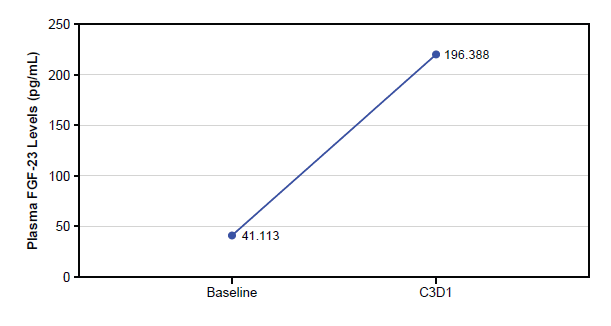
**

**(B)**


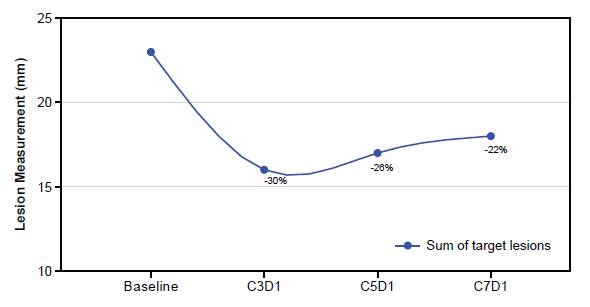


Abbreviations: C, cycle; D, day; FGF, fibroblast growth factor; FGFR, fibroblast growth factor receptor.

**Supplementary Figure S4.** Anti-tumor activity of tinengotinib depicted in *in vivo* iCC PDX models. CC6204, an iCC PDX model with FGFR2-BICC1 fusion, was used in the study. Tumor-bearing mice were orally dosed with vehicle, tinengotinib (15 mg/kg), or futibatinib (40 mg/kg) once daily, whereas gemcitabine hydrochloride (120 mg/kg) was injected intraperitoneally once a week. Tumor volumes (**A**) and body weights (**B**) are presented as geometric mean ± standard error of the mean (*n*=6).

A


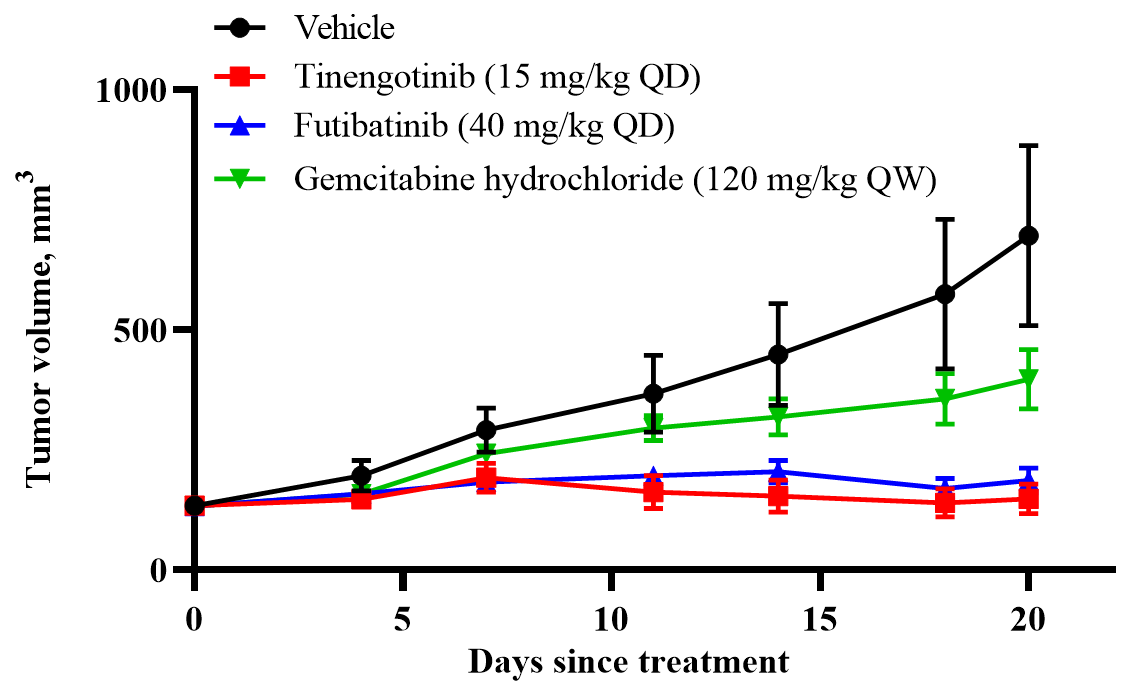


B


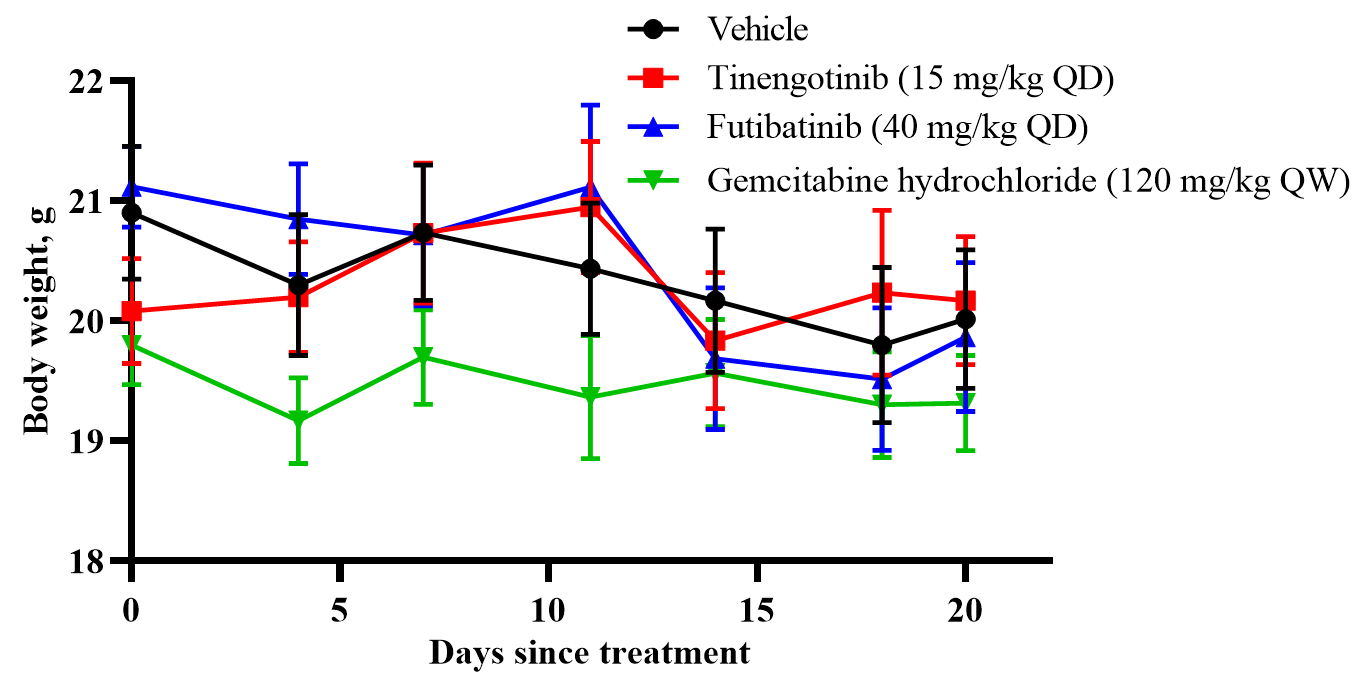


Abbreviations: iCC, intrahepatic cholangiocarcinoma; PDX, patient-derived xenograft; QD, once daily; QW, once weekly.
